# Supplementary material for: Synthetic ECG signal generation using generative neural networks
Source: PLoS One. 2025 Mar 25;20(3):e0271270. doi: 10.1371/journal.pone.0271270 (PMC11936209; doi:10.1371/journal.pone.0271270)
Supplement: Attachment — Submitted filename: plosone_rebutal_rev1.docx [file pone.0271270.s001.docx]

**Reviewer#1, Concern # 1:**

1. Regarding the proposed GANs network, the author **must** provide details about the used training optimization method and if they can provide a comparison between different fine-tuning results using the used optimization techniques.

**Author response:**

Thank you for bringing this to our attention. We added a specific section on the hyperparameter settings.

Typically, to fine-tune hyperparameters in a *classification task*, usually the hyperparameter space is grid-searched and the set of hyperparameters with the lowest metric (accuracy, precision, recall, F1, …) is selected as the best set of hyperparameters.

However, in a generative task idealistically the following procedure should be followed: ***(i)*** hyper-parameter space is defined. ***(ii)*** The performance metric is specified (DTW, Fréchet, visual inspection etc.) and ***(iii)*** the space is grid-searched exhaustively to find the best set of hyperparameters by minimizing the metric. This is practically impossible for cases with large datasets because it would be computationally very expensive, particularly for this study because one single full run (training + evaluation of metrics calculations) for each model takes around 24 hours, and there are numerous hyperparameters in each model and moreover there are 5 models altogether. Probably that was the reason that none of the closely related works to our work has mentioned anything about the hyperparameter fine-tuning in their paper (please refer to Table 3 in paper). So, for the sake of a fair comparison, we treated all models *equally*, and accepted some general suggestions from Radford et al. (2016) and applied them to all models uniformly. Gulrajani et al. (2017) and Golany et al. (2019) both used the same recommendations. The objective of our study was to see how well models can generate realistic synthetic ECG beats, and we believe in this way all models are compared in an equal opportunity situation.

We added a section (Hyperparameter Setting) to the paper describing the hyperparameter settings of the model.

**Author action:** We updated the manuscript by ….

The following section has been added to the paper:

**“Hyperparameter Settings**

In all the models, the number of epochs is 30 with a batch size of 9. The optimizer used is ADAM with β1 and β2 equal to 0.5 and 0.999 respectively. The latent variable of dimension of 100 is used at the generator input. Binary cross-entropy is used as the loss function. Gaussian Normal Distributions with μ of 0 and σ of 0.02 are used for parameter initialization. In model 04 which is a hybrid model of VAE and GAN, the loss generator function is a weighted sum of the adversarial loss and the L1 loss between the decoded beats and real beats.”

**Reviewer#1, Concern # 2:**

2. Add more Figure to show the generated ECG beats especially for the main 5 classes and labelled them.

**Author response:** In this study the focus was solely on Normal beats and all the models were trained accordingly. In our future study we plan to generate 6-7 classes in a conditional and non-conditional GAN setting.

**Author action:** We updated the manuscript by ….

**Reviewer#1, Concern # 3:**

3. Add the equations for performance evaluations.

**Author response:**

Mathematical formulations of the proposed methods 1 through 5 are mentioned in the paper. Also, mathematics behind DTW and Fréchet distance functions have been added and explained very briefly.

**Author action:** We updated the manuscript by ….

The following section has been added:

**Similarity Measures (Distance Functions)**

Currently, there is a lack of consensus on the best evaluation metric for performance of the generative models [22] and researchers mostly resort to the subjective expert-eye evaluation. In general, a distant function DF has a scalar output which quantifies the proximity (or distant) between its two input beats:

$$DF(x, y) : R^{256} x R^{256} \to R$$

***Dynamic Time Warping Measure (DTW)***

DTW belongs to a family of measures known as “elastic dissimilarity measures” and it works by optimally aligning (warping) the time scale in a way that the accumulated cost of this alignment is minimal [23]. It constructs a cost matrix D based on the two time-series being compared, x and y. The elements of matrix D are defined, by a recurrent formula:

$$Di,j = f(xi, yj) + min\{Di-1,j ,Di,j-1,Di-1,j-1\}$$

For i = 1, . . . ,M and j = 1, . . . ,N where M and N are the lengths of the two time series. The local cost function f(., .), also called sample dissimilarity function, is usually the Euclidean distance. The final DTW value typically corresponds to the total accumulated cost, i.e. $dDTW(x, y) = DM,N$ . [23]

***Fr´echet Distance Measure***

If P = (u1, u2, . . . , up) and Q = (v1, v2, . . . , vq) are two time series, a coupling L between P and Q is defined as the set of the links:

(ua1 , vb1 ), (ua2 , vb2 ), . . . , (uam, vbm) (6)

such that $a1 = 1$, $b1 = 1$, $am = p$ and $bm = q$, and for all $i = 1, . . . , q$, $ai+1 = ai$ or $ai+1 = ai + 1$ and $bi+1 = bi$ or $bi+1 = bi + 1$. The length $\parallel L\parallel$ of the coupling L is defined as the longest (maximum Euclidean distance) in the link L:

$$\parallel L\parallel= max d(uai , vbi )$$

$$i=1,...,m$$

(7)

then the Fréchet distance between P and Q is defined as [24]:

$\delta dF = min\{\parallel L\parallel$| L is a coupling between P and Q} (8)

***Euclidean Distance Measure***

The Euclidean distance between two time series, $P = (u1, u2, . . . , up)$ and $Q = (v1, v2, . . . , vq)$ is defined as:

$$d(P,Q) = \sqrt{{(u1 - v1)}^{2} + ・ ・ ・ + {(un - vn)}^{2}}$$

(9)

Fr´echet distance function fulfills all the properties required by metric spaces (e.g., commutative, triangle property, . . .) and can be used as a metric. However, DTW and Euclidean measures do not satisfy the triangle property and are not a metric as required by metric spaces.”

**Reviewer#1, Concern # 4:**

4. I think that authors should make the data available for the public using IEEE DataPort so others can use it to evaluate their algorithms for detection of fake ECG beats.

**Author response:**

Codes are available on GitHub to enable interested users to generate synthetic beats: <https://github.com/mah533/Synthetic-ECG-Generation---GAN-Models-Comparison>

Additionally, codes will be available on IEEE DataPort as well

**Author action:** We updated the manuscript by ….

Codes are available now on IEEE DataPort

**Reviewer#1, Concern # 5:**

5. Add a table the compare the proposed method with methods in and must include more recent techniques and research and organized based on the number of beats.

**Author response:**

A set of comparison tables with the most related recent works has been prepared (Tables 1, 2 and 3). We also designed a new experiment to show the efficacy of synthetic data augmentation on classification performance.

**Author action:** We updated the manuscript by ….

The following sections have been added:

“**Efficacy of Synthetic ECG Augmentation**

A simple experiment has been designed to show the efficacy of the augmentation. A subset of the MIT-BIH Arrhythmia dataset is selected which contained only two classes: N (Normal Sinus Beat) and L (Left Bundle Block Branch Block). Then, a state-of-the-art classifier (EcgResNet34) is trained on ***(i)*** a balanced binary real dataset (L: 6455 and N: 6457) and ***(ii)*** then the dataset is artificially imbalanced (L: 6460 and N: 500) so that the classifier performs poorly on classification. And finally, ***(iii)*** the imbalanced dataset is balanced back again (L: 6458 and N: 6454) by augmenting with

synthetically generated beats. The classifier is trained on each of these three training sets and the classification metrics and confusion matrices are compared with each other in all the three cases. The test set (unseen data) is the same in all three cases (L: 1607 and N: 1609). The classifier used is ResNet34 [25] which is a 34-layer model and is the state-of-the-art in classification of images (2D). It incorporates residual building blocks following the residual stream logic: $F(x) + x$. Each building block is comprised of two 3 × 3 convolutional layers where the residual stream, x, goes directly from the input to the outlet of the block which prevents deterioration of training accuracy in deeper models [25]. This classifier is pretrained on the ImageNet dataset (more than 100, 000 images in 200 classes). We used its 1D implementation [26] to classify the heartbeats”

“**Efficacy of Augmentation**

Comparing the results in Tables 15 and 14 shows that the augmentation of the imbalanced dataset with synthetically generated beats can improve the classification drastically, almost as in real balanced dataset Table 13. Same trend is noticeable from confusion matrices Tables 16 a), b) and c).”

Tables 13, 14, 15 and 16 are added which show the efficacy of synthetic data augmentation.

**Reviewer#1, Concern # 6:**

6. Add a plots for the developed models not only tables.

**Author response:**

Thank you so much for bringing this to our attention. The paper really needed this section. Graphical representations of all models have been added (Fig. 2 to 6)

**Author action:** We updated the manuscript by ….

Graphical representations of models (figures 2 to 6) have been prepared and added to the paper.

***Reviewer #2:*** The manuscript is well organized, and the idea is interesting. My main concern is that the applicability of the approach in realistic application. Specifically, I suggest to design extra experiments to compare the model evaluational performance with generated ECG sample compared to real ECG sample, for example in ECG arrhythmia classification tasks.

**Author response:**

Thank you very much for bringing this to our attention. An extra experiment has been designed and implemented specifically to show the efficacy that the augmentation of synthetic data can have on the classification performance, specifically when the dataset is imbalanced.

First, a balanced dataset (real) is used to train a classifier (ResNET) and metrics are found. Then, the dataset is intentionally Imbalanced so that the classifier performs poorly. In the last step, the imbalanced data set is augmented with synthetically generated ECG signals and the performance metrics and confusion matrices have been found and compared. The improvements in all metrics are obvious.

**Author action:** We updated the manuscript by ….

The following sections have been added:

“**Efficacy of Synthetic ECG Augmentation**

A simple experiment has been designed to show the efficacy of the augmentation. A subset of the MIT-BIH Arrhythmia dataset is selected which contained only two classes: N (Normal Sinus Beat) and L (Left Bundle Block Branch Block). Then, a state-of-the-art classifier (EcgResNet34) is trained on ***(i)*** a balanced binary real dataset (L: 6455 and N: 6457) and ***(ii)*** then the dataset is artificially imbalanced (L: 6460 and N: 500) so that the classifier performs poorly on classification. And finally, ***(iii)*** the imbalanced dataset is balanced back again (L: 6458 and N: 6454) by augmenting with

synthetically generated beats. The classifier is trained on each of these three training sets and the classification metrics and confusion matrices are compared with each other in all the three cases. The test set (unseen data) is the same in all three cases (L: 1607 and N: 1609). The classifier used is ResNet34 [25] which is a 34-layer model and is the state-of-the-art in classification of images (2D). It incorporates residual building blocks following the residual stream logic: $F(x) + x$. Each building block is comprised of two 3 × 3 convolutional layers where the residual stream, x, goes directly from the input to the outlet of the block which prevents deterioration of training accuracy in deeper models [25]. This classifier is pretrained on the ImageNet dataset (more than 100,000 images in 200 classes). We used its 1D implementation [26] to classify the heartbeats”

“**Efficacy of Augmentation**

Comparing the results in Tables 15 and 14 shows that the augmentation of the imbalanced dataset with synthetically generated beats can improve the classification drastically, almost as in real balanced dataset Table 13. Same trend is noticeable from confusion matrices Tables 16 a), b) and c).”

Tables 13, 14, 15 and 16 are added which show the efficacy of synthetic data augmentation.
